# Diagnostic value of combined ultrasound contrast and elastography for differentiating benign and malignant thyroid nodules: a meta-analysis

**DOI:** 10.1038/s41598-024-63420-1

**Published:** 2024-06-01

**Authors:** Funing Liu, Yihan Wang, Yu Xiong, Xin Li, Jun yao, Hao Ju, Fu Ren, Luwei Zhang, Hongbo Wang

**Affiliations:** 1 School of First Clinical College, Shenyang Medical College, Shenyang, People’s Republic of China; 2 School of Public Health, Shenyang Medical College, Shenyang, People’s Republic of China; 3 School of Stomatology, Shenyang Medical College, Shenyang, People’s Republic of China; 4 School of Forensic Medicine, China Medical University, Shenyang, People’s Republic of China; 5 Shengjing Hospital of China Medical University, Shenyang, People’s Republic of China; 6 Department of Human Anatomy, School of Basic Medicine, Shenyang Medical College, Shenyang, People’s Republic of China; 7 Key Laboratory of Human Ethnic Specificity and Phenomics of Critical Illness in Liaoning Province, Shenyang, People’s Republic of China; 8 Key Laboratory of Phenomics Research, No.146, Huanghe North Street, Shenyang, 110034 Liaoning People’s Republic of China; 9 242 Hospital of Shenyang Medical College, Shenyang, People’s Republic of China

**Keywords:** Contrast-enhanced ultrasound, Ultrasonic elastography, Thyroid nodules, Meta-analysis, Computational biology and bioinformatics, Endocrinology

## Abstract

The diagnostic value of contrast-enhanced ultrasound combined with ultrasound elastography for benign and malignant thyroid nodules is still controversial, so we used meta-analysis to seek controversial answers. The PubMed, OVID, and CNKI databases were searched according to the inclusion and exclusion criteria. The literature was selected from the establishment of each database to February 2024. The QUADAS-2 tool assessed diagnostic test accuracy. SROC curves and Spearman's correlation coefficient were made by Review Manager 5.4 software to assess the presence of threshold effects in the literature. Meta-Disc1.4 software was used for Cochrane-Q and *χ*^2^ tests, which be used to evaluate heterogeneity, with *P*-values and *I*^2^ indicating heterogeneity levels. The appropriate effect model was selected based on the results of the heterogeneity test. Stata18.0 software was used to evaluate publication bias. The diagnostic accuracy of contrast-enhanced ultrasound combined with ultrasound elastography for benign and malignant thyroid nodules was evaluated by calculating the combined sensitivity, specificity, positive likelihood ratio, negative likelihood ratio, DOR, and area under the SROC curve. A total of 31 studies included 3811 patients with 4718 nodules were analyzed. There is no heterogeneity caused by the threshold effect, but there is significant non-threshold heterogeneity. Combined diagnostic metrics were: sensitivity = 0.93, specificity = 0.91, DOR = 168.41, positive likelihood ratio = 10.60, and negative likelihood ratio = 0.07. The SROC curve area was 0.97. Contrast-enhanced ultrasound and elastography show high diagnostic accuracy for thyroid nodules, offering a solid foundation for early diagnosis and treatment.

*Trial registration*. CRD42024509462.

## Introduction

A thyroid nodule is a localized mass or tumor within the thyroid tissue, a discrete lesion formed by abnormally growing localized thyroid cells, which may be single or multiple. Approximately 60% of adults have at least one thyroid nodule, indicating their widespread prevalence^1^. Due to the widespread use of ultrasonography, many untouchable thyroid nodules are found in unrelated accidental examinations^2^. However, only about 5% of thyroid nodules are proven to be malignant^3^. Therefore, accurate diagnosis of benign and malignant thyroid nodules is essential for subsequent treatment^4^. Ultrasonography and fine needle aspiration biopsy (FNAB) are fundamental in diagnosing thyroid nodules^5^. Accurate diagnosis can greatly alleviate the depression and anxiety associated with diagnostic errors^6^. In order to achieve a unified evaluation of thyroid nodules and reduce the influence of subjective factors, some scholars proposed and established the thyroid imaging, reporting and data system (TI-RADS) was constructed in 2009^7^. For the diagnostic criteria of this system, thyroid nodules are graded 2–6 according to different malignant rates. Ultrasound-based TI-RADS released by the American College of Radiology (ACR) in 2017 provides the basis for generating systematic reports. The system ensures that all nodules can be classified and provide clear diagnostic recommendations (biopsy, follow-up, and no intervention) with high specificity. It is the current guideline for the evaluation and interpretation of disease-oriented imaging studies. A large number of studies have performed retrospective analysis to explore that the use of ACR TI-RADS can reduce the false negative rate of malignant nodules^8^.

The gold standard diagnostic method for thyroid nodules is histopathological findings from surgical biopsy. Yet, this approach is invasive, costly, and time-intensive^9^. FNAB examination is considered an excellent diagnostic tool for evaluating thyroid nodules due to its high sensitivity, specificity, economy, and wide acceptance. It achieves accurate results in approximately 80% of cases^1,10^. However, FNAB is an invasive technique that, although minimally invasive, is not accepted by all patients.

In view of the initiative of ultrasound examination, the different experience of diagnostic physicians, and the overlapping ultrasound signs of benign and malignant thyroid nodules, some nodules that should be diagnosed as TI-RADS grade 3 or grade 5 were classified as TI-RADS grade 4. Therefore, the use of conventional ultrasound alone in the diagnosis of thyroid nodules is becoming more and more rare^11^. Contrast-enhanced ultrasound (CEUS) and surround elastography (SE) are ultrasound techniques widely used in examining and diagnosing thyroid nodules in recent years. The principle of contrast-enhanced ultrasound is to diagnose benign and malignant thyroid nodules by applying high-frequency sound waves to the human body and entering its echo to generate images. Ultrasound contrast agent can enhance the contrast and clarity of ultrasound images, which is conducive to the diagnosis of benign and malignant thyroid nodules. Contrast agents are observed earlier in thyroid cancer nodules compared to benign hyperplastic nodules and adenomas^12^. Contrast-enhanced ultrasound is a new technique, and its potential in diagnosing malignant thyroid nodules is unclear, such as thyroid cancer^13^. Ultrasonic elastography is an imaging technique for measuring tissue elasticity. Ultrasound elastography is an ultrasound technique that evaluates the nature of the lesion by measuring the elastic properties of the tissue. According to the different physical quantities measured, it can be divided into real-time tissue elastography (RTE), shear wave elastography (SWE), acoustic radiation force impulse imaging (ARFI), and so on^14^. The hardness of the tissue is quantified in the form of a color map to evaluate the nature of the thyroid nodule. The cancer tissue is more rigid and solid than the thyroid parenchyma or benign thyroid nodules. The advantages of contrast-enhanced ultrasound and ultrasound elastography include being non-invasive, cost-effective, widely accessible, and bedside applicable^15,16^.

Contrast-enhanced ultrasound and ultrasound elastography are common ultrasound techniques for the diagnosis of thyroid nodules. The thyroid gland is located on the body surface, and the measurement of nodule hardness is easily affected by respiration and large blood vessel pulsation in the neck^17^. For that reason, there is a certain bias in evaluating the malignant risk of thyroid TI-RADS 4 nodules based only on SWE results. Owing to TI-RADS 4 nodules have different proliferation stages and the nodules themselves are diverse, their imaging features are complex and overlapping. Thus, there are also errors in the evaluation of nodules by CEUS. Trimboli et al*.*^18^ showed that the sensitivity and specificity of contrast-enhanced ultrasound in the diagnosis of thyroid nodules were more than 80%. Zhou et al*.*^19^ showed that the sensitivity of ultrasound elastography in the diagnosis of benign and malignant thyroid nodules was 72.26% with specificity was 95.35%. It can be seen that contrast-enhanced ultrasound and ultrasound elastography have high sensitivity for the diagnosis of thyroid nodules. Hence we speculate that contrast-enhanced ultrasound combined with ultrasound elastography can greatly improve the diagnostic accuracy of thyroid nodules, so that it can approach the diagnostic accuracy of the gold standard.

However, there is no consensus yet on the diagnostic accuracy of contrast-enhanced ultrasound and ultrasound elastography for thyroid nodules. This could be attributed to the ongoing development of these technologies. Meta-analysis offers more comprehensive, accurate, and reliable conclusions by synthesizing the results of multiple studies. Therefore, based on the histopathological diagnosis as the reference standard, we intend to conduct a meta-analysis of the diagnostic value of contrast-enhanced ultrasound combined with ultrasound elastography for benign and malignant thyroid nodules and conduct a comprehensive evaluation, aiming to explore the diagnostic value of CEUS combined with SE in the differential diagnosis of benign and malignant thyroid nodules.

## Methods

This study adheres to the PRISMA guidelines^20^. As this meta-analysis synthesizes existing studies, it did not require ethical approval.

### Literature retrieval

To identify studies eligible for inclusion in this meta-analysis, two online English databases (PubMed and OVID) and one online Chinese database (CNKI) were searched. The search time was from the establishment of the database to February 2024. We included all types of publications, peer-reviewed articles, dissertations, and conference papers, with language restricted to English and Chinese. To maximize recall and precision, we used MeSH terms, free-text keywords, and Boolean operators in our searches. English search terms included ‘thyroid nodule,’ ‘ultrasonic elastography,’ ‘ultrasonography,’ ‘contrast-enhanced ultrasound,’ and their Chinese equivalents were used as well.

### Inclusion and exclusion criteria

Articles that met the following criteria were considered for inclusion: (i) randomly selected continuous samples and provide the number of thyroid nodules; (ii) prospective or retrospective study with a paired design; (iii) combined diagnosis of contrast-enhanced ultrasound and ultrasonic elastography; (iv) clearly explain the diagnostic criteria for pathological examination; (v) provide full text and can obtain a 2 × 2 table or included data, including the number of true positives, the number of false positives, the number of false negatives, and the number of true negatives.

Exclusion criteria were as follows: (i) the number of cases in the literature was less than 20; (ii) the content and information of the articles were incomplete; (iii) reviews, commentaries, letters, and Meta-analyses; (iv) duplicate publications.

### Data extraction and migration risk assessment

Based on the inclusion and exclusion criteria, two researchers independently extracted data from eligible studies. Disagreements were resolved through discussion until consensus was achieved. Data extracted encompassed the first author's name, publication year, study country, instruments used, diagnostic methods, sample sizes, numbers of thyroid nodules, and counts of true positives, false positives, false negatives, and true negatives.

Two researchers independently assessed the risk of bias in the included studies using the Quality Assessment of Diagnostic Accuracy Studies-2 (QUADAS-2). Bias risk was evaluated using ‘yes,’ ‘no,’ and ‘uncertain’ for methodological quality, and ‘high,’ ‘low,’ and ‘unclear’ for bias risk across domains including case selection, index tests, reference standards, and flow and timing.

### Data analysis

Spearman correlation coefficients of the logarithm of sensitivity versus the logarithm of (1-specificity) and summary receiver operating characteristic (SROC) curves were calculated using Meta-DiSc1.4 to test for the presence of threshold effect heterogeneity in the included literature. The Cochrane-Q test and χ^2^ test were used to test the non-threshold effect heterogeneity of the diagnostic odds ratio (DOR) of each study, and I^2^ assessed the magnitude of heterogeneity, *P* < 0.1 indicated that there was non-threshold effect heterogeneity in the studies, I^2^ ≤ 25% was small heterogeneity, 25% < I^2^ < 50% was moderate heterogeneity, and I^2^ < 50% was moderate heterogeneity, and I^2^ ≤ 0.1 indicated that there was non-threshold effect heterogeneity. The degree of heterogeneity and I^2^ ≥ 50% suggest a significant degree of heterogeneity among the included studies, and a random effects model should be used. Meta-regression using Meta-DiSc1.4 was used to find the causes of heterogeneity after meta-regression, subgroup analyses were performed, and sensitivity analyses were carried out by Stata18.0 software. Sensitivity, specificity, positive likelihood ratio, negative likelihood ratio, DOR, and their corresponding 95% CIs were combined using Meta-DiSc1.4 software, and *P* < 0.05 indicated that the difference was statistically significant. Forest plots of sensitivity, specificity, and combined subject work characteristics (SROC) curves were drawn. The area under the curve (AUC) and Q-value were analyzed by the SROC curve.

We used Deeks linear regression analysis of Stata18.0 software to evaluate publication bias. The data distribution was significantly asymmetric, and *P* < 0.05 was statistically significant, suggesting that there was publication bias.

By excluding each of the included studies one by one, we observed changes in heterogeneity and pooled effect sizes after the removal of a particular study to assess the stability of the results. If there is no significant change in the combined effect size after removing each study, the results are stable and credible. If the results are quite different after analysis, it shows that the sensitivity is high and the stability of the results is low.

## Results

### Literature screening results

**Figure 1:**
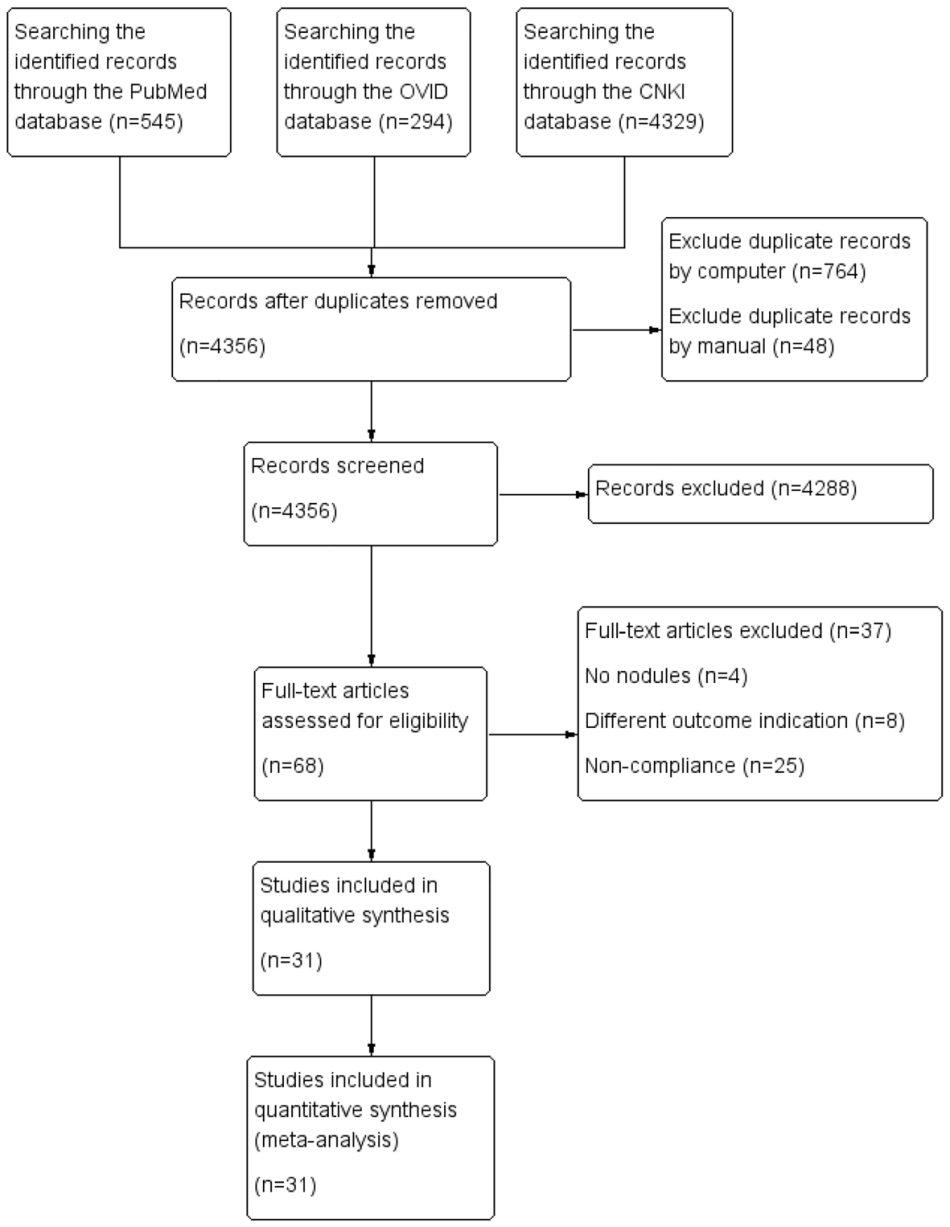
Flow chart of literature screening.

Initially, we screened 5168 articles, comprising 545 from PubMed, 294 from OVID, and 4329 from CNKI. After removing 810 duplicate articles using EndNote software, 4191 articles that were significantly unrelated to the patient population and diagnostic methods of this study were further excluded by reading the title and abstract. In addition to this, there are 99 reviews, comments, letters, and meta-analyses. Following initial screening, 65 articles met the inclusion criteria and were available in full text. We excluded four articles for not specifying the number of thyroid nodules, eight for differing outcome indicators, and 25 for not meeting other inclusion criteria. A total of 34 articles were excluded after rescreening. Ultimately, 31 articles were included for analysis: 24 in Chinese and 7 in English. All the articles included in the analysis used a combination of CEUS and elastography to diagnose thyroid nodules. The analysis encompassed 3811 patients and 4718 nodules. (Fig. 1).

### Characteristics of included studies

The detailed characteristics of the included studies (Table 1) included the first author, year of publication, country, instrument, diagnostic method, sample size, number of thyroid nodules, and the number of true positive, false positive, false negative, and true negative. What we need to make a special explanation here is that all the ultrasound contrast agents in the study used sulfur hexafluoride microbubbles (SonoVue), the mechanical index used the default index of each device, and all articles used strict TIC quantitative criteria for the diagnosis of thyroid nodules.

**Table 1:** Detailed characteristics of the included studies.

Author	Year	Country	Apparatus	Diagnosis	Total	Nodules	TP	FP	FN	TN
Li^25^	2011	China	Acuson Sequoia 512, Hitachi 6500 HV +	CEUS + SE	82	82	33	7	1	41
Lin^26^	2012	China	Acuson Sequoia 512, Hitachi 6501 HV +	CEUS + SE	82	164	66	14	2	82
Deng^27^	2013	China	Siemens Acuson S2000	CEUS + ARFI	90	110	34	11	2	63
Deng^28^	2014	China	Siemens Acuson S2000	CEUS + ARFI	146	175	54	4	2	115
Lu^29^	2015	China	Siemens Acuson S2000	CEUS + SE	50	50	21	2	1	26
Kang^30^	2015	China	Philips iU 22	CEUS + SE	86	98	38	2	2	56
Liu^31^	2015	China	HI VISION 900, PHILIPS HDI4000	CEUS + SE	128	160	64	4	4	88
Wang^32^	2015	China	Philips iU 22	CEUS + SE	164	328	132	28	4	164
Mo^33^	2016	China	Mylab Twice	CEUS + SE	250	291	23	12	20	195
Chen^34^	2016	China	Philips iU22 Xmatrix, Aixplorer	CEUS + SWE	253	319	127	13	9	170
He^35^	2016	China	Philips iU 22	CEUS + SE	96	152	55	4	12	81
Sui^36^	2016	China	Philips iU 22, Semensi Acuson S2000	CEUS + RTE	97	107	63	2	3	41
Wang^37^	2016	China	LOGIQ E9	CEUS + SE	150	159	68	3	7	81
Hu^38^	2016	China	Siemens Acuson S2000	CEUS + SE	120	150	60	3	4	83
Me^39^	2017	China	Siemens Acuson S2000	CEUS + SE	49	60	18	4	1	37
Wu^40^	2017	China	Philips	CEUS + SE	100	122	22	1	1	98
Li^41^	2017	China	Philips IU Elite	CEUS + SE	60	60	27	4	6	23
Xu^42^	2017	China	PHILIPS iE Elite, AlokaProsound 风范 F75	CEUS + SE	24	50	17	4	2	27
Xu^43^	2018	China	Philips iU 22	CEUS + SE	146	218	81	13	2	122
He^44^	2018	China	GE E9, Siemens Acuson S2000	CEUS + ARFI	83	88	27	6	2	53
Deng^45^	2018	China	Logic-E9	CEUS + RTE	76	88	27	5	0	56
Zhao^46^	2019	China	Philips iU 22	CEUS + SE	307	367	140	49	10	168
Zuo^47^	2019	China	Philips iU 22	CEUS + SE	73	114	50	1	2	61
Lin^48^	2019	China	Esaote MyLab 90	CEUS + SE	226	236	53	19	9	155
WangJ^49^	2020	China	Aixplorer	CEUS + SWE	185	196	78	11	3	104
Yao^50^	2020	China	Philips	CEUS + SE	116	148	60	4	2	82
Wang^51^	2020	China	ND	CEUS + SE	70	55	17	1	1	36
Du^52^	2021	China	Siemens S3000	CEUS + SE	80	80	33	0	5	42
Zhang^53^	2021	China	ND	CEUS + SE	130	152	63	5	5	87
Huang^54^	2021	China	Esaote MyLab 90	CEUS + SE	200	221	139	17	12	53
He^55^	2022	China	GE Logiq E9	CEUS + SE	92	118	84	7	1	26

*CEUS* contrast-enhanced ultrasound; *SE* strain elastography; *RTE* real-time elastography; *SWE* shear wave elastography; *ARFI* acoustic radiation force impulse; *ND* not mentioned.

### Quality assessment

**Figure 2:**
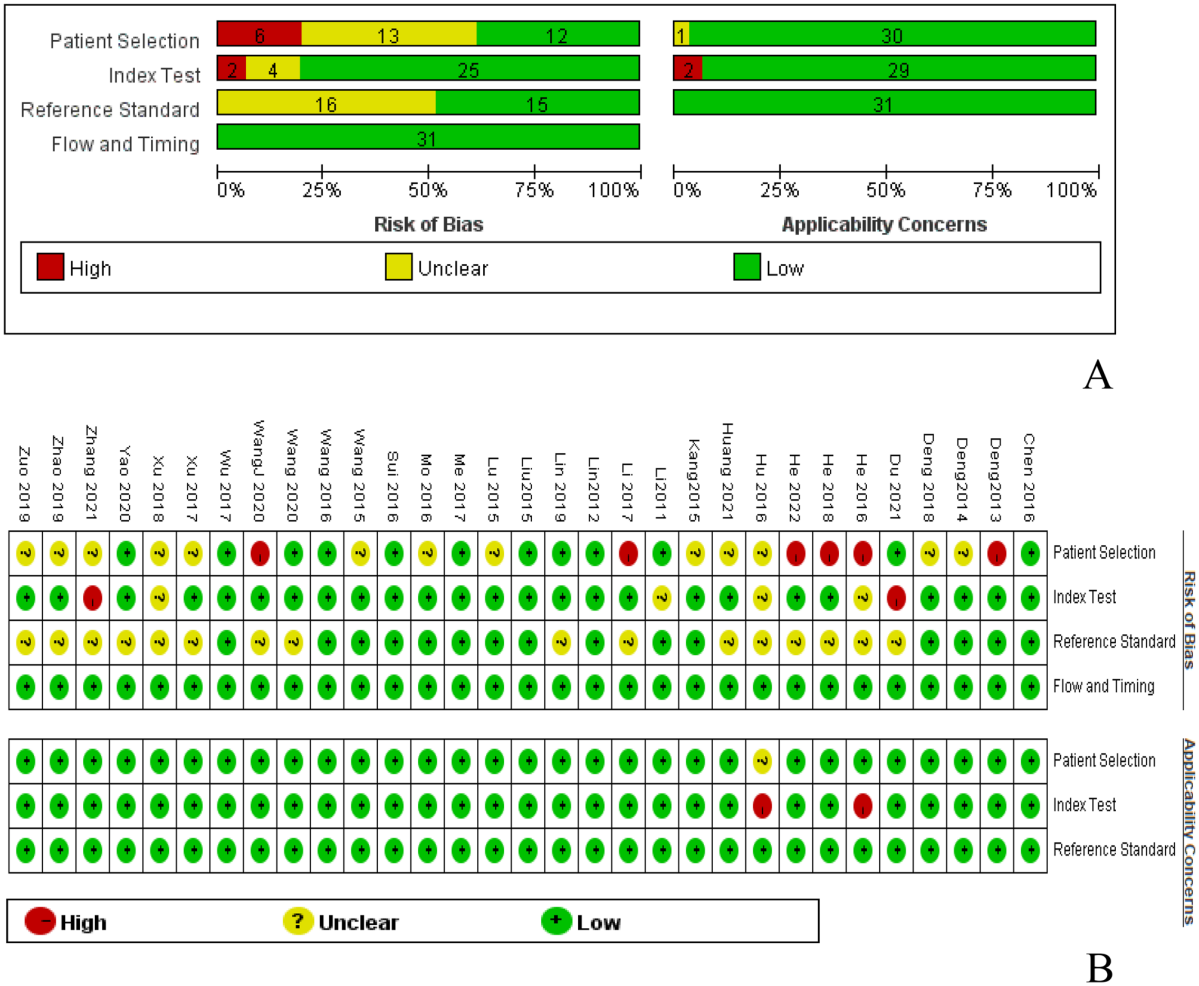
Methodological quality of the 31 included studies. (**A**) Methodological quality summary (**B**).

All the included literature was evaluated according to the QUADAS-2 evaluation tool. Evaluation results indicated high quality in case flow progression and clinical applicability. However, the literature revealed a low level of quality in the continuous selection of cases, the threshold setting of evaluation tests, and the blind implementation of gold standard interpretation (Fig. 2).

### Statistical results

#### Heterogeneity test

**Figure 3:**
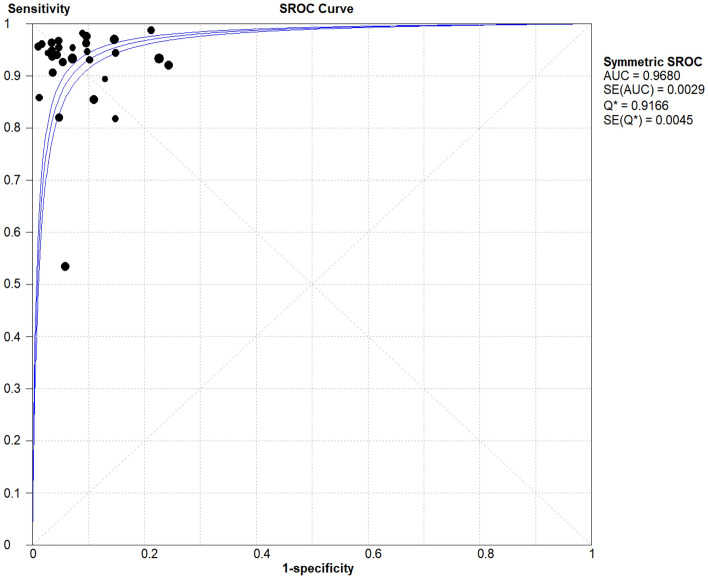
Comprehensive DOR of the 31 included studies.

The SROC curve analysis revealed that study data points do not exhibit a ‘shoulder-arm’ distribution (Fig. 3), with a Spearman correlation coefficient of r = 0.037 (*P* = 0.844), suggesting an absence of threshold effect heterogeneity. However, when using the Cochrane-Q test and χ2 test to test the heterogeneity of the diagnostic odds ratio (DOR) of each study, *P* < 0.10, I^2^ = 63%, suggesting that there was significant heterogeneity caused by non-threshold effects among the included literature.

#### Combining statistical results

**Figure 4:**
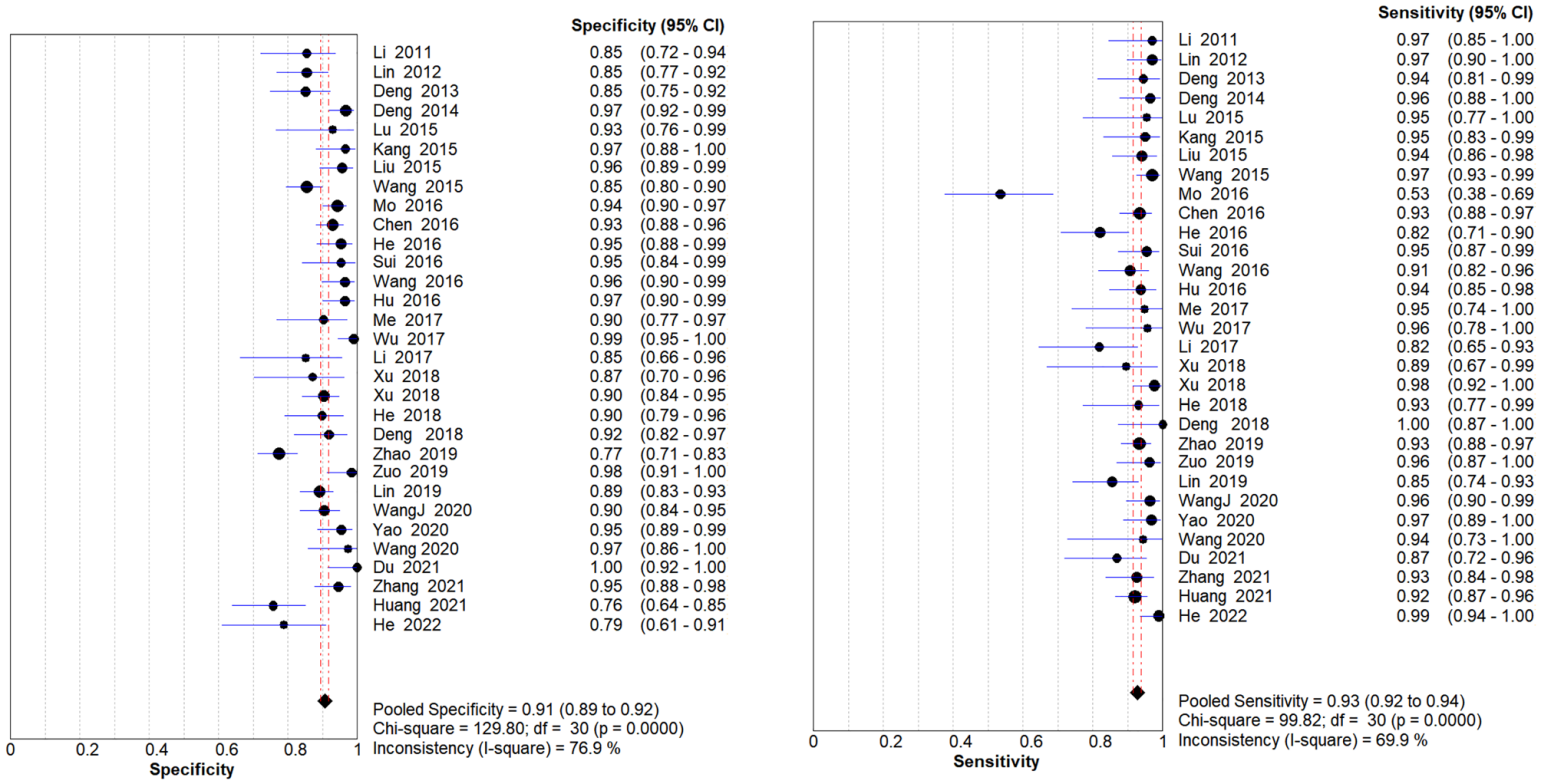
Forest plots of sensitivity (SEN) and specificity (SPE) with corresponding 95% CIs.

**Figure 5:**
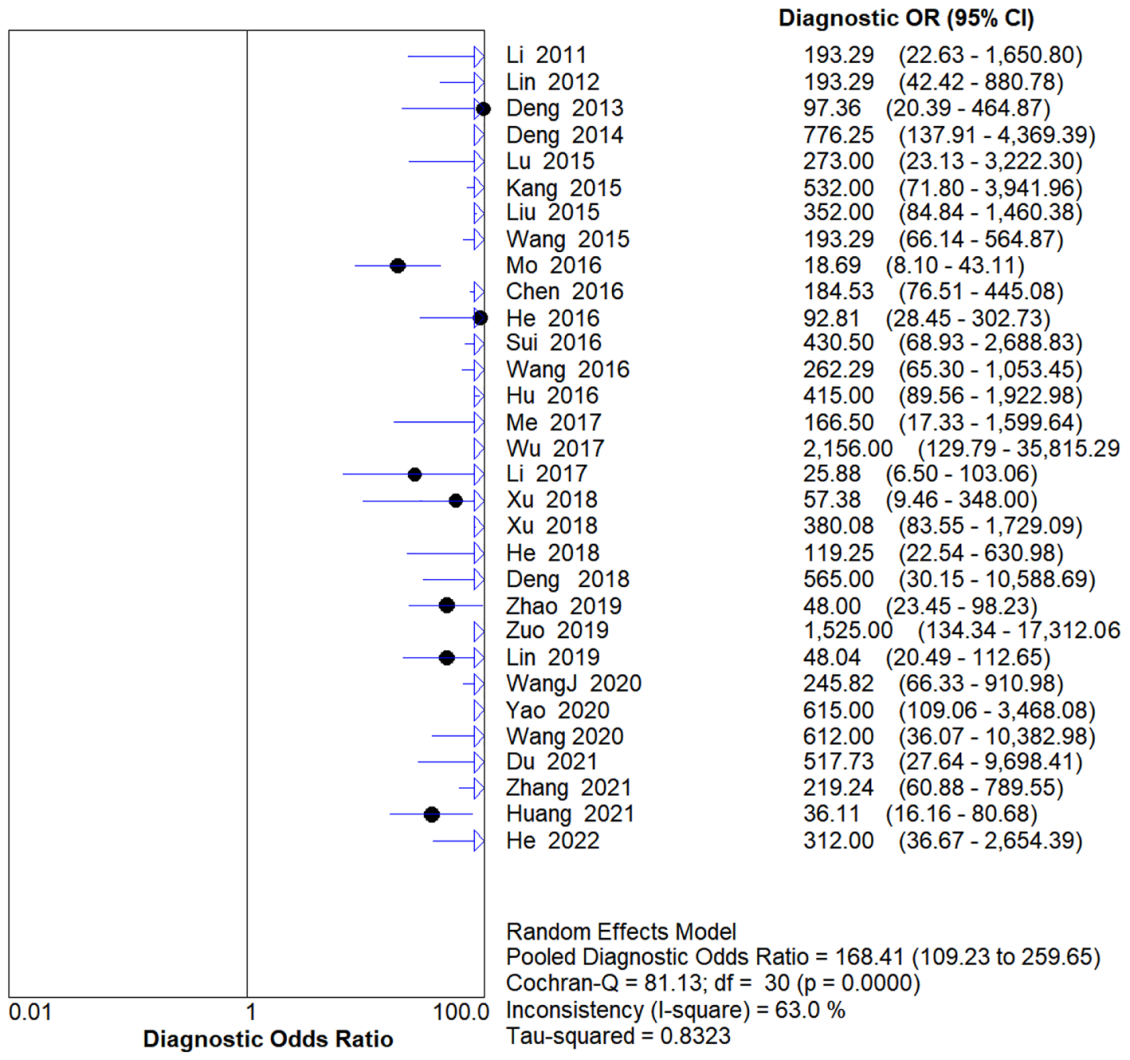
Forest plots of the diagnostic OR (DOR) with corresponding 95% CIs.

Given the results of the heterogeneity test, the random effect model was used to analyze the combined effect size. The pooled sensitivity was 0.93 (95% CIs 0.92–0.94), the pooled specificity was 0.91 (95% CIs 0.89–0.92) (Fig. 4), the DOR was 168.41 (95% CIs 109.23–259.65) (Fig. 5), the pooled positive likelihood ratio was 10.60 (95% CIs 8.26–13.60), the pooled negative likelihood ratio was 0.07 (95% CIs 0.05–0.11), and the area under the SROC curve (AUC) was 0.97. The Q * index was 0.92. It shows that contrast-enhanced ultrasound combined with ultrasound elastography has high diagnostic efficiency for benign and malignant thyroid nodules.

### Subgroup analysis

The results of the heterogeneity test showed significant heterogeneity caused by the non-threshold effect among the studies. According to the factors that may produce heterogeneity, they were divided into different subgroups, such as the number of devices used in diagnosis, diagnostic methods, and sample size. The results of the subgroup analysis showed that the subgroup using two instruments for diagnosis had higher sensitivity than the subgroup using one instrument, and the subgroup did not mention the number of instruments used. Compared with contrast-enhanced ultrasound combined with real-time tissue elastography, contrast-enhanced ultrasound combined with shear wave elastography has higher sensitivity, specificity, and diagnostic odds ratio in the diagnosis of benign and malignant thyroid nodules. The combined sensitivity and specificity of each subgroup are shown in Table 2.

**Table 2:** Subgroup analysis results.

Subgroup	Number of studies	Combined SEN(95%CIs)	Combined SPE(95%CIs)	DOR(95%CIs)
Apparatus				
1	22	0.92 (0.98–1.00)	0.90 (0.89–0.92)	163.24 (93.34–285.31)
2	7	0.95 (0.92–0.97)	0.91 (0.88–0.93)	189.19 (110.01–325.35)
ND	2	0.93 (0.85–0.97)	0.95 (0.90–0.98)	261.04 (81.24–838.82)
Diagnosis				
CEUS + RTE	26	0.92 (0.91–0.94)	0.90 (0.89–0.92)	164.92 (100.02–271.94)
CEUS + SWE	5	0.95 (0.92–0.97)	0.92 (0.89–0.94)	199.31 (111.64–355.82)
Total				
≥ 100	15	0.93 (0.91–0.94)	0.91 (0.89–0.92)	163.33 (87.31–305.53)
< 100	16	0.93 (0.91–0.95)	0.91 (0.89–0.93)	164.48 (97.86–276.46)

*CEUS* contrast-enhanced ultrasound; *RTE* real-time elastography; *SWE* shear wave elastography; *ND* not mentioned.

### Publication bias test

Deeks linear regression analysis was used to evaluate publication bias. The results showed that T = 1.46 (*P* = 0.16) and the data distribution did not show significant asymmetry, indicating no evidence to support significant publication bias in the included literature.

### Sensitivity analysis

**Figure 6:**
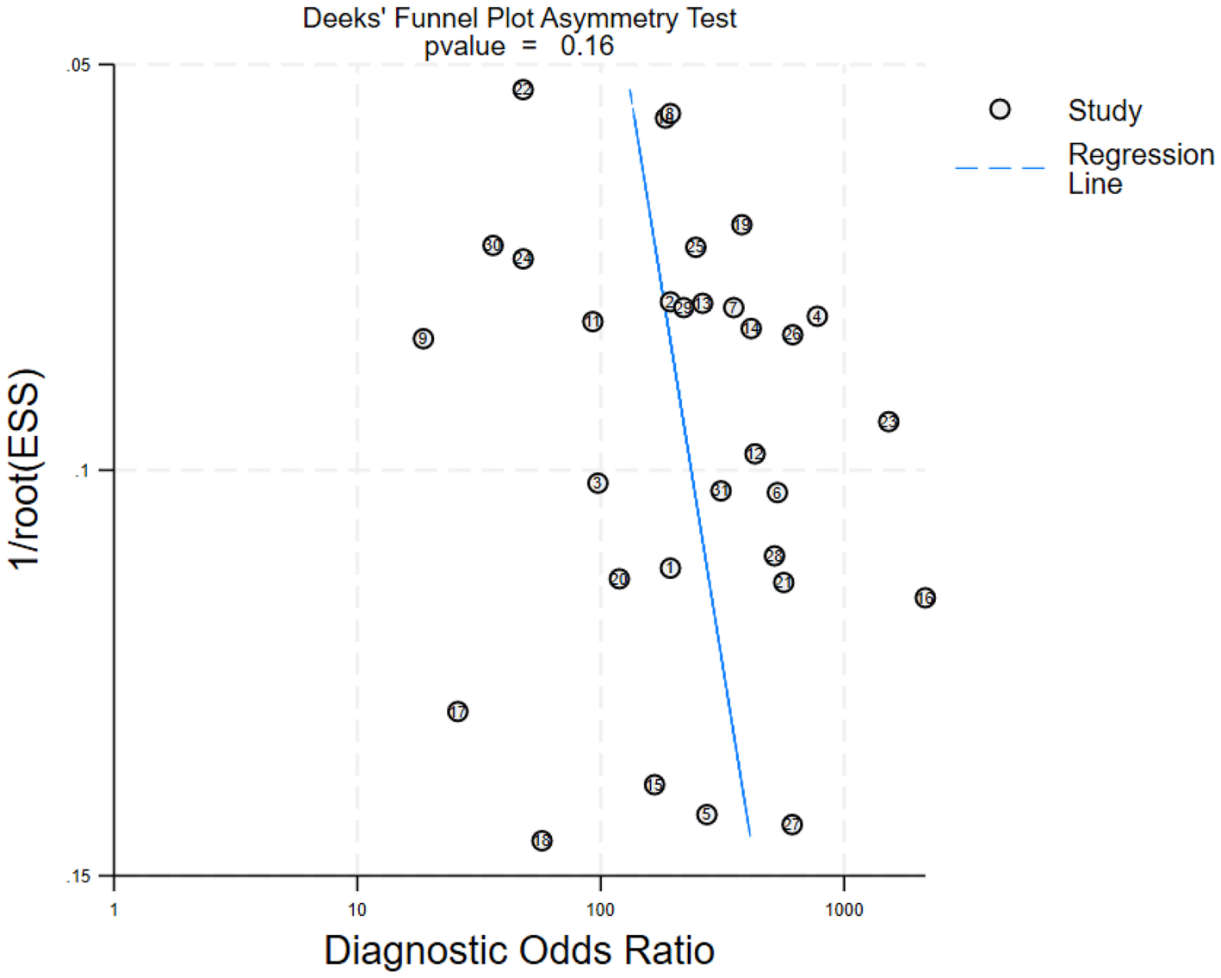
The funnel plot of publication bias.

The sensitivity analysis was used to test the impact of individual studies on the combined statistics in the included studies. After each included study was removed, in turn, there was no significant change in the combined sensitivity, specificity, and 95% CIs, indicating that the results of this meta-analysis were stable (Fig. 6).

## Discussion

This study included 31 articles, encompassing 3811 patients and 4718 nodules. The results showed that the combined sensitivity of contrast-enhanced ultrasound combined with ultrasound elastography in the diagnosis of benign and malignant thyroid nodules was 0.93, the combined specificity was 0.91, the combined diagnostic odds ratio was 168.41, the combined positive likelihood ratio was 10.60, the combined negative likelihood ratio was 0.07, and the area under the SROC curve was 0.97, indicating that contrast-enhanced ultrasound combined with ultrasound elastography has a high diagnostic value for the differential diagnosis of benign and malignant thyroid nodules.

This meta-analysis summarizes several original studies on the differential diagnosis of benign and malignant thyroid nodules by contrast-enhanced ultrasound combined with ultrasound elastography in recent years and uses a random effect model to incorporate the sensitivity, specificity, and other effect indicators of the included studies and overcomes the shortcomings of the small sample size of a single study. The meta-analysis method objectively evaluates the value of contrast-enhanced ultrasound combined with ultrasound elastography in the differential diagnosis of benign and malignant thyroid nodules. A 2018 meta-analysis of 21 studies demonstrated enhanced diagnostic outcomes with this combination, advocating for broader dissemination^21^. We updated this meta-analysis by including ten recent studies. It will be more comprehensive to ascertain the role of contrast-enhanced ultrasound combined with ultrasound elastography in the diagnosis of thyroid nodules. In this study, a comprehensive literature search was conducted, strict inclusion and exclusion criteria were established, and the methodological quality of the 31 papers included was moderate. After statistically pooled the effect sizes of the included papers, we found that the combined sensitivity of all the studies was 0.93, the combined specificity was 0.91, and the area under the SROC curve was 0.97, which suggests that ultrasonography combined with ultrasound elastography has a high degree of effectiveness in the diagnosis of benign and malignant thyroid nodules. This indicates that ultrasonography combined with ultrasound elastography has high diagnostic accuracy for the identification of benign and malignant thyroid nodules. It can be used as a co-diagnostic method because of its advantages of being non-invasive and inexpensive^22,23^. Contrast-enhanced ultrasound is a recognized non-invasive and economical diagnostic tool^15^. Ultrasound elastography has the advantages of being objective, repeatable and less affected by the operator^24^. It can be examined at the bedside for critically ill patients or those who cannot go to the ultrasound room. Contrast-enhanced ultrasound combined with ultrasound elastography has a high accuracy rate, which can provide clinicians with reasonable and effective treatment measures for different types of patients. Ultrasound elastography combined with CEUS can make up for the shortcomings and deficiencies in single diagnosis, and has high diagnostic efficiency. It can provide more diagnostic information from different angles, which is helpful to detect malignant nodules as soon as possible, and provide an effective basis for the formulation of clinical treatment plans. It is beneficial for patients to receive targeted treatment plans as soon as possible and promote the prognosis of the disease. The results of the Meta-analysis by Hu et al*.*^4^ on the diagnostic potential of real-time elastography (RTE) and shear wave elastography (SWE) for identifying benign and malignant thyroid nodules showed that the accuracy of RTE for diagnosis of thyroid nodules was better than that of SWE, but in this study, interestingly, we found that the diagnostic accuracy of CEUS combined with SWE for benign and malignant thyroid nodules is higher than that of CEUS combined with RTE. Moreover, if ultrasonography and ultrasound elastography are performed in both devices, the diagnostic sensitivity is significantly increased.

After exploring the sources of heterogeneity through Meta-regression and subgroup analyses, we found that sample size may be the main factor leading to heterogeneity. The subgroup analysis results showed that using two instruments could improve the accuracy of diagnosis of benign and malignant thyroid nodules. This may be due to the differences between the instruments, and the combined use of two instruments reduces the random errors of one instrument in the diagnostic process. The combined sensitivity and specificity of ultrasonography combined with shear wave elastography in diagnosing thyroid nodules were 0.95 and 0.92, both higher than that of ultrasonography combined with real-time strain elastography, which may be related to the fact that shear wave elastography is more sensitive and accurate in assessing the nature of the nodules quantitatively by using data indexes such as "Young's modulus."

The primary limitation of this meta-analysis is inherent to its methodology, including a restricted literature base and a lack of detailed instrument classification. Due to the limited number of literature included in the study, this study has not yet classified each instrument included in the study, nor has it further studied the diagnostic accuracy of elastography combined with contrast-enhanced ultrasound for benign and malignant thyroid nodules other than real-time strain elastography and shear wave elastography. Future research should broaden the search scope, detail diagnostic instruments into subgroups, and include various types of ultrasound elastography to enhance diagnostic accuracy analysis. In addition, the included literature is only in English and Chinese, which may cause language deviation. It is seemingly unreasonable that 24 study in Chinese and 7 in English were include in our meta-analysis. This may be due that doctors in other countries are inclined to use FNAB than CEUS or other ultrasound techniques to evaluate thyroid nodules, and believe that in many cases where FNAB is still needed for diagnosis, there is no need to use CEUS as an expensive technique.

## Conclusions

This meta-analysis assessed the diagnostic accuracy of combining contrast-enhanced ultrasound with ultrasound elastography for differentiating benign from malignant thyroid nodules. Results indicate that the use of any ultrasound elastography technique with contrast-enhanced ultrasound significantly enhances diagnostic sensitivity and specificity. Consequently, we recommend this combination as a non-invasive diagnostic method that not only improves diagnostic accuracy but also minimizes tissue damage from unnecessary biopsies, lowers clinical risks associated with fine needle aspiration, and supports the early diagnosis and treatment of malignant thyroid nodules.

## Data Availability

The datasets used and/or analyzed during the current study are available from the corresponding author on reasonable request.

## Abbreviations

CEUS: Contrast-enhanced ultrasound
RTE: Real-time elastography
SWE: Shear wave elastography
DOR: Diagnostic odds ratio
CIs: Confidence interval
